# Supplementary material for: Learning curve and surgical outcome of robotic assisted colorectal surgery with ERAS program
Source: Sci Rep. 2022 Nov 29;12:20566. doi: 10.1038/s41598-022-24665-w (PMC9709162; doi:10.1038/s41598-022-24665-w)
Supplement: Supplementary file 2 — Supplementary Information 2. [file 41598_2022_24665_MOESM2_ESM.zip › RAL Raw data-Ñ[▒K/063/HS_ñw╝╨░O▒Kñσ.pdf]

正式摘要(電子簽章：000000212342716)		
W52 / W52 ☐英文標題(注意:無英文姓名-請補病人基本檔!)	☐【讀取】憑證	

臺中榮民總醫院
出院病歷摘要

轉入號 : 轉出號 :

[redacted]

**(8)**流水編號	
**(9)**入院日期	110/03/10 (2021) 直腸外科 W52 - 161
**(10)**轉科日期	
**(11)**出院日期	110/03/15 (2021) 住院天數計 5 日

診斷

- 入院 :Rectal Ca, 5 cm FAV, Adenocarcinoma, moderately differentiated
- 出院 :Rectal Ca, 5 cm FAV, Adenocarcinoma, moderately differentiated, s/p Robotic LAR on 2021/03/11

主訴

- boody stool for months

病史

- This 52 y/o male with a hx of

  1. Rectal cancer, Adenocarcinoma, moderately differentiated, of rectum, 5 cm FAV, w/ paraaortic retroperitoneal LN 1.5 cm
  2. HBV carrier for 40 years
  3. DM just under OHA
  4. hyperlipidemia

  This time, he complained of boody stool for months. There was also change of defecation habits, intermittent diahrrea and constipation. There was no body weight loss or general weakness in recent half year. UGI and LGI scope were done and showed chronic gastritis and Rectal Ca, 5 cm FAV, adenocarcinoma, moderately differentiated. Under the impression of Rectal Ca, 5 cm FAV, Adenocarcinoma, he was admitted for Robotic LAR.

體檢發現

- PS 70%,
  1100302 Side Effect: Dry mouth(Grade1)
  1100310 No side effect
  - General appearance :
    - easy looking , conscious clear , without cardiopulmonary distress
    - Vital signs自動帶入date 體溫(BT) 脈搏(P) 呼吸(R) 血壓(BP) 身高(BH) 體重(BW) BMI
      20210310 35.9℃ 93次/分 20次/分 142/87mmHg 172cm 83.8kg 28.3
    - Vital signs: B.P. 142/87 mmHg , P.R. 93 /min , R.R. 20 /min , B.T. 35.9 ℃
  - HEENT :
    - Eyes: no anemic conjunctiva, no icteric sclera, normal EOM
    - ENT: no otorrhea, no rhinorrhea, no erythematous mucosa, no injected tonsil, no deformity
  - Neck: supple, freely movable, trachea midline,no deformity, no engorged jugular vein , no palpable thyroid or lymph node enlargement
  - Chest:
    - Inspection: Grossly normal, symmetric and fully expansion, no accessory muscle used, no operation scar, no spider angiomata, no gynecomastia
    - Palpation: no palpable mass or nodule, no subcutaneous emphysema, no tenderness
    - Ausculation: clear breathing sound

- Heart:
  - Palpation: no thrill, no heave
  - Percussion: normal heart size
  - Ausculation: regular heart beat, normal S1, S2, no gallop, no murmur
- Extremities: No deformity, full ranged of motion without limitation, no clubbing finger or nail cyanosis, no pitting edema, no ulcer or pigmentation, no obvious skin lesion, no varicose vein, no bone, joint or muscle tenderness

CRS CONDITION

- Karnofsky performance status: (100) Normal, no complaints, no evidence of disease
- PE of Abdomen:
- CEA: 3.67 ng/ml
- Imaging Studies:
- Colonoscope report: Rectal Ca, 5 cm FAV, adenocarcinoma, moderately differentiated
- Figures:
  1. colon & rectum

手術日期及方法

- 手術日期及方法20210311 Robot Assisted Low Anterior Resection + LOOP ILEOSTOMY

住院治療經過

- After admission, Robotic LAR was done smoothly on 2021/03/11. On ERAS protocol, he tried liquid diet on 03/12 then BLAND II diet on 03/13. We discharged the patient on 03/15 with stable condition.

合併症

一般檢查

- 累積報告：

DATE	TIME	ALB	TP	BIL.T	BIL.D	ALKP	AST	ALT	LDH	CHOL	TG	HDL-C	LDL	UA	NH3	AMY
1100310	2049									178	245	34	114	6.5		
1100311	1508	4.2		0.46	0.14		51	35								

DATE	TIME	WBC	RBC	HGB	HCT	MCV	PLT	BLST	NEUT	BAND	SEG	LYM	MON	EOS	BAS
1100311	1509	9070	5.05	14.1	40.2	79.6	218		92.5			3.9	3.3	0.0	0.3

DATE	TIME	NA	K	CL	CA	BUN	CREAT	Mg	P	CRP	ESR	PCT	LIPASE	LACTATE
1100310	2049									0.408				
1100311	1508	137	3.8	99	8.8	9	0.89							

DATE	TIME	GLUCOSE	PAB	TRF	ZN	CU	KETONE	CK	CKMB	PT-P	PT-C	APTT-P	APTT-C	TRO-T
1100310	2051	253												
1100311	1508	268												

特殊檢查

病理報告

其它

出院時情況

- 15.計劃性出院

出院指示

藥名	劑量	劑量單位	用法	頻次	數量	數量單位	現況	天數	單位
Mag. Oxide tab	2	TAB	PO	TID	42	TAB	TH	7	天
Ultracet tab	1	TAB	PO	Q6H	28	TAB	TH	7	天

出院計劃

主治醫師 2062F 蔣鋒帆 / 住院醫師 2325G 劉佳樺

正式摘要(電子簽章：**000000212342716**)

W52 / W52

doctorRank:B
